# Breaking down the barriers: fMRI applications in pain, analgesia and analgesics

**DOI:** 10.1186/1744-8069-2-30

**Published:** 2006-09-18

**Authors:** David Borsook, Lino R Becerra

**Affiliations:** 1 P.A.I.N. Group, Brain Imaging Center, Department of Psychiatry, McLean Hospital, Belmont, MA, USA; 2 Athinoula Martinos Center for Biomedical Imaging, Department of Radiology, Massachusetts General Hospital, Boston, MA, USA

## Abstract

This review summarizes functional magnetic resonance imaging (fMRI) findings that have informed our current understanding of pain, analgesia and related phenomena, and discusses the potential role of fMRI in improved therapeutic approaches to pain. It is divided into 3 main sections: (1) fMRI studies of acute and chronic pain. Physiological studies of pain have found numerous regions of the brain to be involved in the interpretation of the 'pain experience'; studies in chronic pain conditions have identified a significant CNS component; and fMRI studies of surrogate models of chronic pain are also being used to further this understanding. (2) fMRI studies of endogenous pain processing including placebo, empathy, attention or cognitive modulation of pain. (3) The use of fMRI to evaluate the effects of analgesics on brain function in acute and chronic pain. fMRI has already provided novel insights into the neurobiology of pain. These insights should significantly advance therapeutic approaches to chronic pain.

## Background: 'O (Chronic) Pain Miserum'

**Figure 1:**
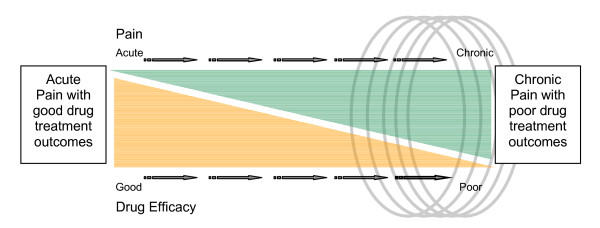
**The problem with chronic pain**. Therapy for acute pain (e.g., acute inflammation, trauma, post-surgical pain) is overall excellent. However, in chronic pain (e.g., neuropathic pain, fibromyalgia, complex regional pain syndrome), therapy is poor. This group thus falls into a zone (circles) of "therapeutic failure" or "therapeutic impasse" where multiple therapies are tried with overall little success. Functional imaging appears poised to open up new approaches to the understanding of chronic pain conditions. An improved basic understanding of the mechanisms underlying chronic pain is likely to suggest new avenues for the development of novel pharmacotherapies.

At least two major hurdles remain in the treatment of chronic pain. The first is that no objective test for pain currently exists. A blood test, genetic marker or psychophysical measure would greatly improve diagnosis of chronic pain. The second is the lack of an "antibiotic equivalent" (i.e., drugs with high sensitivity and specificity) for the treatment of chronic pain subtypes (e.g., neuropathic pain). Controlled trials of drug efficacy indicate that, on average, the most effective drugs of different classes have similar efficacy (around 30% greater than placebo) across neuropathic conditions [1-3]. Analgesic use is dictated by both efficacy and adverse side effects and side effects can sometimes take precedence over efficacy [4]. A lack of controlled trials for other methods of pain treatment (interventional, psychological, physical therapy) makes it difficult for physicians to evaluate these possible therapies. As a result, chronic pain treatment is difficult, and physicians and patients often resort to using multiple treatments simultaneously or sequentially in the effort to achieve pain relief. Unfortunately, even a combined therapeutic approach frequently offers little benefit (Figure 1).

Recent advances in functional imaging have revolutionized our concept of central process of pain. Indeed, it seems that we are on the verge of using this technology to reach a fundamental new understanding of clinical pain, particularly chronic pain (defined as pain lasting for more than 6 months). Subdivision of chronic pain syndromes into chronic neuropathic pain (e.g., phantom pain, post-herpetic neuralgia), chronic nociceptive pain (e.g., arthritis, migraine), and a group comprising very poorly understood categories of pain (e.g., fibromyalgia, depression-induced pain, or complex regional pain syndrome) has not clarified mechanistic processes. Even classification of chronic pain types based on clinical disease (e.g., cancer pain, diabetic pain) has not proved very helpful in understanding the mechanisms underlying chronic pain. Recently a mechanistic approach to defining pain has been suggested in which specific pain phenotypes such as shooting pain, burning pain, and allodynia can be applied across pain types such as neuropathic pain. [5]. However, this approach is based primarily on an understanding of peripheral nerve and spinal cord processing. Functional imaging has already redefined chronic pain as a degenerative disease, and has shed some light on complex diseases such as fibromyalgia [6]. Since brain responses are the final common pathway in behavioral responses to pain (unconscious and conscious), we believe that the application of functional imaging will allow us to categorize pain conditions in an objective manner and to better understand the underlying circuitry and identify targets for a new generation of analgesics [7,8].

fMRI measures neural activity by an indirect evaluation of changes in blood flow in capillary beds [9]. A number of approaches including block design [10], event-related [11], and percept-related. [12] paradigms, have been applied to fMRI studies of physiological, clinical and pharmacological aspects of pain and analgesia. Application of baseline measures of spontaneous pain have allowed the "basal state" to be evaluated.) [13]. Issues pertaining to the validity of fMRI in pain and analgesic measures have been presented elsewhere [14].

"***To consider only the sensory features of pain, and ignore its motivational and affective properties, is to look at only part of the problem, not even the most important part at that." ***This statement of Melzack and Casey's [15] was an early recognition of these aspects of pain, but their importance is now widely accepted. The ability to use fMRI to image the whole brain at the same time and to use powerful algorithms to segregate functional circuits allows us to begin to elucidate the CNS processes underlying affective and motivational components of pain. It also allows a broader window to observe potential CNS sites of drug action. Our understanding of 'difficult' disease states (e.g., fibromyalgia or depression-related pain), the placebo response, emotional responses (e.g., empathy), and acupuncture will clearly be influenced by new insights into how emotional circuitry in the brain functions in pain states and in responses to analgesics. [16]. In this paper, we review the contribution of fMRI to the understanding of acute and chronic pain, its use in surrogate models and for evaluation of endogenous pain systems including the placebo response, and its potential use as an objective measure of analgesic efficacy. The approach we have taken to summarize the new advances has been to provide an overview for each domain (e.g., Acute and Chronic pain, Endogenous Systems etc.) and summary tables that focus on specific areas within each domain (e.g., Chronic pain: Neuropathic, Fibromyalgia etc.). Studies listed are predominantly from peer-reviewed journals (Data Sources: Medline) or data from our own lab presented at Society for Neuroscience and/or in press. We attempted to include primary examples on specific entities of CNS processing as defined with fMRI that are related to pain, analgesia and analgesics.

## Advancing our Understanding of CNS Mechanisms in Acute and Chronic Pain through fMRI

fMRI of Physiological Pain – a new understanding of brain regionsinvolved in pain processing in humans

Much of the work in fMRI of pain has utilized thermal stimuli (contact peltier thermodes or laser) to activate pain circuits. Other types of stimuli, including electrical and mechanical (pressure) have not been as extensively used either in the intact system or where sensitization has been experimentally induced (see Table 3). The accumulation of data has begun to identify brain regions involved in pain processing – from peripheral ganglia to central limbic and brainstem structures previously only implicated in animal studies in the preclinical literature (see Table 1).

**Table 3:** Examples fMRI Studies of Surrogate Models of Pain

**STUDY FOCUS**	**REFERENCE**	**MAJOR FINDINGS**	**COMMENT**
**Experimental Allodynia**			
Capsaicin induced secondary hyperalgesia	Baron et al., 1999 [81]	9 Subjects. Capsaicin injection induced secondary mechanical hyperalgesia. Painful mechanical stimulation produced activation in prefrontal cortex >> than in nonpainful mechanical stimulation	First fMRI study of capsaicin induced hypersensitivity. Activation in prefrontal cortex = attention and cogniitvie changes (e.g., planning).
Brainstem activation by capsaicin	Zambreanu et al., 2005 [82]	Heat-capsaicin model used. Punctate mechanical stimuli applied to region of secondary hyperalgesia. Stimuli in hyperalgesic vs. control region showed activation in contralateral brainstem, cerebellum, bilateral thalamus, contralateral SI and SII, middle frontal gyrus, parietal association cortex and brainstem (cuneiformis, superior colliculi, PAG.	First evaluation of contribution of brainstem in central sensitization.
Cognitive influences on hyperalgesia	Wiech et al., 2005. [83]	Capsaicin-induced heat hyperalgesia results in frontal and medial prefrontal cortex, insula, and cerebellum. Activity in medial prefrontal cortex and cerebellum modulated by cognitive task.	Study addresses interaction between motivational and cognitive functions and may provide some basis for evaluating similar changes in chronic pain.
Capsaicin allodynia	Maihofner et al., 2004. [39]	Region of allodynia produced by capsaicin and thermal kindling. Brush to normal skin results in SI, parietal association cortex, SII bilaterally, contralateral insula. Brush to allodynic skin resulted in some overlap to those observed for control in addition to inferior frontal cortex, and ipsilateral insula.	Subtraction (unaffected vs. affected skin) indicates that mechanical allodynia in regions that include SI, parietal association cortex, inferior frontal cortex, and insula.

**Definitions of terms in Table 3**:

Allodynia – pain to a normally non-noxious stimulus.

Central Sensitization – increased sensitivity or excitability in central pain pathways as a result of increase in sensitivity/activation of these neural systems

Hyperalgesia – An increased response to a stimulus that is normally painful

Secondary Hyperalgesia – pain outside of the area of the primary injury.

**Table 1:** Examples of Contributions by fMRI on the understanding Brain Regions activated by Acute Pain

**STUDY FOCUS**	**REFERENCE**	**MAJOR FINDINGS**	**COMMENT**
**Peripheral Nerve**			
Trigeminal Ganglion (TG)	Borsook et al., 2003. [63]	Somatotopic activation in the trigeminal ganglion	Measures of the peripheral nervous system may be evaluated using fMRI
**Dorsal Horn**			
Trigeminal Nucleus (TN)	DaSilva et al., 2002. [20]	Somatotopic acitivation in the TN	Study reports that pain spinal cord brainstem systems can be defined and somatotopically evaluated.
**Brainstem**			
Periaqueductal Gray (PAG)	Becerra et al., 2001 [17]	Both increases (early) and decrease (late) of activation may correlate with ascending and descending (modulatory) components of functioning within this structure.	The PAG is a 'core' structure in understanding how the brain modulates pain, both in placebo and in the effects of analgesics, particularly opioids.
Right Cuneus	Fulbright et al., 2001 [64]	Cold pressor induced pain produced activation in a number of regions including the frontal lobe and the cuneus.	Measures of affective components of cold pain.
Brainstem NucleiCuneiformis, parabrachial, PAG, red nucleus	Dunckley et al., 2005 [65]	Activation to somatic and visceral pain.	A big step forward in measures of brainstem measures of pain in humans. Marked similarities in the two processes were observed at a brainstem level.
**Subcortical Gray Regions**			
Emotion CircuitryAccumbens, SLEA, Amygdala, Hippocampus, Hypothalamus, Oribitofrontal Cortex	Becerra et al., 2001 [17]	Acute pain activates circuitry that is commonly associated with reward. Emotional circuitry is activated ahead of sensory circuitry	The first demonstration that reward circuitry can be mapped in acute pain.
Amygdala	Seymour et al., 2005 [21]	Termination of pain (rewarding) activates the amygdala	The significance of understanding the brain systems to natural reward (i.e., pain relief).
Putamen	Bingel et al., 2004 [66]	15 Subjects. Laser evoked pain to foot or hand produced contralateral somatotopic organization in putamen	Clear activation in putamen by pain indicative of potential role in emotional or motor processing of pain.
Accumbens (NAc)	Aharon et al., 2006 [18]	Acute noxious (but not non-noxious) stimuli activate the NAc. Within the structure, different signals may indicate functional processing within the 'core' and 'shell' of the structure.	A number of regions have different functional components (e.g., amygdala, PAG) and the ability to dissect apart these within a set paradigm will contribute further to mechanistic functions of pain processing in humans.
**Cortical Regions**			
Anterior Cingulate(aCG)	Becerra et al., 1999 [10], 2001 [17]	One of the first fMRI studies to demonstrate differential aCG activation in the structure.	Differentiation of sensory vs. emotional components of aCG function.
Hippocampus (Hi)	Ploghaus et al., 1999 [27], 2000 [67], 2001 [28]	Hippocampal activation correlates with anxiety/	Papers address a specific function of the hippocampus in pain and further show a correlation with insula activity.
Insula (I)	Brooks et al., 2005. [19]	Somatotopic organization in the insula defined	The insula has been a bit of an enigma. Based on preclinical work, human work seems to support the notion that the insula is receives thermal information from the ventromedial nucleus of the thalamus (VMpo) specific thermal stimuli
Oribtofrontal Cortex (GOb)	Rolls et al., 2003. [68]	Effects of pleasant and painful touch to hand. Oribitofrontal activation > pleasant or pain vs. neutral. SI less activated by pleasant and pain than neutral touch. Regional differences in aCG to pleasant (rostral aCG) and pain (posterior dorsal). Brainstem (e.g., PAG) activated by all 3 touch stimuli.	Clear dissociation between sensory and emotional systems to 'reward' and aversion.
Somatosensory Cortex	Bingel et al., 2004 [66]	Painful laser stimuli applied to hand and foot produced somatotopic organization in contralateral and ipsilateral SI cortex.	Laser stimuli can provide stimuli without tactile components.

Some of these regions are part of well-defined pain circuits (e.g., PAG, parabrachial nuclei) while for others such as the nucleus accumbens, their specific role in pain processing is not well understood [17,18]. Studies have reported specificity of somatotopic organization of structures outside of the primary somatosensory cortex involved in pain processing in humans; these such as the insula [19] and the trigeminal system [14,20]. Indeed, the results of human imaging help focus attention on specific regions, including the nucleus accumbens (involved in emotional salience), the insula (involved in specific interpretation of noxious stimuli) or the amygdala (involved in fear), opening new vistas for understanding how the human brain evaluates pain. The ability to evaluate activity and organize active regions into neural circuits that subserve specific pain/analgesia functions (i.e., sensory, emotional, autonomic, endogenous analgesic circuits) is a step forward. A number of studies have already begun this task including segregation of function within a structure (e.g., PAG [17] and NAc [18]), defining analgesia as a rewarding process [21], and functional differentiation of activation sites within a particular brain region (e.g., cognitive and affective regions within the anterior cingulate [17]).

What is needed now is to further evaluate these brain areas, some of whose role is newly defined in pain processing in humans, at a functional level. Several new fMRI approaches will aid this effort including techniques that allow definition of large scale systems organization [22]; techniques that define circuits/functional connectivity [23]; and automated parcellation of brain structures [24,25] including the thalamus [26]. Imaging studies have not only unveiled new regions involved in pain processing, but have also contributed new insights into the functioning of these regions in experimental pain. For example, the hippocampus, classically associated with memory, has been shown to be involved in pain-induced anxiety [27-29].

### fMRI of Chronic Pain

Imaging clinical conditions is fraught with issues that make it more challenging, including the fact that it is difficult to assemble a cohort with similar symptoms, duration of disease, medication history, age distribution, etc. Among clinical conditions, chronic pain has a particularly wide spectrum of patient presentations and medical histories. However, studies have begun to evaluate CNS changes that occur in patients with chronic pain (see review by Apkarian and colleagues [30]), including those with neuropathic pain, fibromyalgia, complex regional pain syndrome and visceral pain (Table 2).

**Table 2:** Examples of Contributions of fMRI to the understanding of CNS circuitry underlying Chronic Pain

**STUDY FOCUS**	**REFERENCE**	**MAJOR FINDINGS**	**COMMENT**
**Neuropathic Pain**			
Mechanical Allodynia	Peyron et al., 2004. [69]	Activated regions mirror control network activated by brush, cold to the normal side. Regions activated include SI, SII and insula.	Study tackles an issue of ongoing pain and the problems associated with ongoing background pain.
Back Pain vs. Postherpetic Neuropathy	Apkarian et al., 2004. [34]		A potentially huge step forward in the use of fMRI to differentiate chronic pain subtypes.
Trigeminal Neuropathy	Becerra et al., 2005 [33] Becerra et al., 2006 [70]	V2 neuropathy patients evaluated in a repeat study for mechanical (brush) and thermal (cold and heat) stimuli.	Mechanistic changes in CNS function to specific stimuli.
Chronic Back Pain	Giesecke et al., 2004. [31]	Heterotopic pressure stimulus applied to the thumb activates a number of brain regions.	Generalized increase in pain sensitivity in chronic pain; pain >> in patients than controls for the same pressure stimulus. Equally painful stimuli produced similar brain activations.
Spinal Cord Injury (SCI)	Nicotra et al., 2005 [71]	Seven patients with SCI evaluated including painful stimuli (shock) in an aversive paradigm. Conditioning stimuli produces enhancement of activity in dorsal anterior cingulated, PAG and superior temporal gyrus to conditioning stimuli and attenuation in subgenual aCG, ventromedial prefrontal and posterior cingulated to threat of shock.	The study is able to dissect apart possible changes in the brains of SCI patients, including central sensitization and alterations in affective components of the brain (subgenual aCG) that may be part of a disturbance of affective and autonomic processing.
Unpleasant Odor	Villemure et al., 2005 [72]	Single patient with neuropathic pain where pain increased when exposed to experimental odors in thalamus, amygdala, aCG and SI.	This study may suggest subcortical mechanisms of aversion have a common neural circuitry.
**Complex Regional Pain Syndrome**			
Pediatric CRPS	Lebel et al., 2005. [73]	Pediatric group with relapsing CRPS of lower extremity. Changes to cold most predominant.	Clinical models within particular groups of diagnosis may be used to determine the etiology of more chronic conditions e.g., adult CRPS.
Mechanical Allodynia in Adult CRPS	Maihofner et al., 2005 [40]	Twelve Patients. Pin-prick hyperalgesia activates a number of cortical regions (SI, SII, Insula, aCG, frontal cortex).	Study focus is on cortical regions only and indicates significant changes in functioning in affected vs., unaffected. No control group.
Sympathetically Maintained Pain (SMP)	Apkarian et al., 2001. [74]	Evaluation of stimuli to painful site before and after sympathetic blockade. SMP associated with increased prefrontal, aCG activation and decrease in contralateral thalamus.	Correlates of CNS function shown:ineffective blocks did not change cortical activityplacebo response same as effective block
**Fibromyalgia**			
Primary fibromyalgia (FM)	Cook et al., 2004 [75]	Fibromyalgia compared with control group. Fibromyalgia group more sensitive on psychophysical evaluation. Non-painful stimuli produced greater activation in a number of regions including prefrontal, SMA, insula and cingulated cortices. Pain produced greater activation in the contralateral insula in (FM) patients.	Central changes with increased sensitivity/hyperalgesia are clearly manifest behaviorally and on fMRI. Such insights provide a new approach to understanding a heretofore ill- defined disease.
Catastrophizing	Gracely et al., 2004 [6]	Pressure stimulus applied to the thumb (i.e., heterotopic). SII activation >> in high catasrophizers; contralateral aCG and bilateral lentiform nucleus. This was independent of depression.	Catastrophizing may contribute to the pain state by enhancing the emotional reaction to pain.
**Visceral Pain**			
Functional bowel disorder	Kwan et al., 2005 [76]	Healthy (11) vs. Patients (9) underwent painful rectal distention. Activation in the medial thalamus, hippocampus for pain.	On line ratings of pain responses. – clear differences in emotional circuitry of medial thalamus and hippocampus.
Irritable Bowel Syndrome (IBS) with constipation vs. diarrhea	Wilder-Smith et al., 2004 [77]	Female healthy (10) and IBS (10; 5-constipated and 5-diarrhea) underwent rectal distention and painful heterotopic pain applied to activate DNIC. Significant differences in CNS regions (prefrontal cortex, amygdala, aCG, PAG, Hippocampus) between constipated and diarrhea groups and controls.	Different responses in patient subtypes of IBS suggesting differences in endogenous modulatory systems.
Irritable Bowel Syndrome (IBS)	Mertz et al., 2000 [78]	Healthy (16) vs. patients with IBS (18), IBS patients have increased activation in aCG.	Increased central sensitivity to the same type of stimulus.
Visceral and Cutaneous Hypersensitivity in Irritable Bowel Syndrome (IBS)	Verne et al., 2003 [32]	Rectal cutaneous pain produced increased activation in thalamus and SI, I, aCG, pCG and prefrontal cortex.	The brain is adversely affected in chronic pain – both visceral and cutaneous hyperalgesia produced. These findings provide a window on how we may address treatments for these patients.
Irritable Bowel Syndrome (IBS)	Bonaz et al., 2002. [79]	Rectal pain produced in 11 female subjects with IBS. Activation in insula, amygdala and striatal regions. Greater activation in patients in the insula and frontal regions.	Similar to other studies a more complex alteration in pain processing is present in this group of subjects. Issues of variability in patients are still a concern.
**Chronic Inflammation**			
Vulvar vestibulitis	Pukall et al, 2005. [80]	Allodynia measured in patients (14) and controls (14) age and contraceptive matched.	Similar type of changes in patients with IBS, Fibromyalgia.

A number of important issues are emerging in the evaluation of chronic pain conditions as fMRI and associated imaging technologies become more sophisticated. Chronic pain produces changes that become manifest as alterations in the central nervous system. One may term this "centralization of hyperalgesia" or "centralization of pain". This has been evaluated across diseases including fibromyalgia [6], chronic back pain [31], and irritable bowel syndrome [32]. During functional imaging of fibromyalgia and chronic back pain, enhanced responses to thermal or mechanical stimuli that are applied heterotopically (i.e., away from the actual location of the pain) are present in a number of CNS regions, including non-sensory regions. Differences in specific responses to brush, heat and cold in affected vs. intact regions in patients with neuropathic pain have also been reported [33]. One feature that seems to be of in common with chronic pain patients is significantly greater frontal lobe activation in chronic pain sufferers [30]. This feature, suggests that in chronic pain CNS activity in regions involved in cognitive processing differs between acute and chronic pain. These insights are further complicated by the new and revolutionary recognition that, in chronic pain, neuronal loss occurs in significant pain pathways including the thalamus and the lateral prefrontal cortex [34]. The role of this neurodegeneration in producing either the altered CNS responses or the pain state is not understood.

Maladaptative changes in non-sensory circuits may contribute to the psychological states, including depression, anxiety and amotivation that are often seen in these patients. Thus the study of specific brain regions such as the nucleus accumbens (involved in probability assessments and reward evaluation), the amygdala (involved in orientating to and the memory of motivationally salient stimuli), the hippocampus (involved in evaluating the expectancy of an unknown condition), the prefrontal cortex (involved in cognitive and planning functions around emotional stimuli or regarding rewarding or aversive outcomes) and the anterior cingulated cortex (involved in the rank ordering of the value or salience of the stimulus) may provide new insights into brain functioning in these co-morbid conditions. Such insights should provide immediate benefits to the understanding of the calcitrant nature of chronic pain to therapeutic interventions.

Our increased recognition that multiple neural systems are involved in pain processing and affect pain perception (reward/aversion circuitry, the role of anticipation, neural systems interpreting different pain types albeit at the same intensity, opponent systems and drug effects) suggests that multiple neural circuitries are likely affected in chronic pain. Results published thus far indicate that: (1) Pain intensity is probably not a good marker for changes in chronic pain state. Neural imaging may be able to define a correlation between CNS activation and patient answers to a simple subjective questionnaire that assesses emotional and other components of pain and suffering; (2) Neural systems interpreting components of the pain response (e.g., emotional, empathy, anticipation etc.) are clearly complex, and we still have no understanding of how these may change in the chronic pain condition; and (3) Standards will need to be applied across imaging facilities in order to interpret and compare data across studies.

### fMRI of Human Surrogate Pain Models

Defining valid surrogate models has been a problem in both animal and human models of pain [35-37]. In human studies, mechanical (heat) or chemical (capsaicin) sensitization of skin and testing in primary and secondary regions affected has been used as a surrogate for neuropathic pain (hyperalgesia/allodynia to thermal and mechanical stimuli) [38]. Recently fMRI has been used to evaluate the capsaicin – induced hyperalgesia model (see Table 3). While many of these studies report increased activation in a number of brain regions, some of the more recent studies begin to define the utility of fMRI in dissecting mechanistic changes or insights using this model [39-41]. These studies demonstrate differences in brain activation in response to stimuli of equivalent pain intensity delivered in sensitized vs. non-sensitized state, providing further evidence that pain intensity by itself is probably not a useful measure of the status of the underlying pain processing circuitry [41]. An alternate explanation may be that there are mechanistic differences between these two states. Changes in perceived pain intensity may reflect acute changes in CNS sensory pathways but may not correlate with changes in CNS emotional pathways which may be relevant to an individuals' overall response to pain and may predict future pain conditions. Eventually, fMRI should allow the direct comparison of activation in specific CNS regions in experimental models with the activation seen in patients with neuropathic pain. Comparing such objective measures should allow us to determine where the model differs from the disease and to assess the validity of such models for evaluating potential therapies.

## fMRI studies of Endogenous Analgesia

Endogenous modulatory networks can either facilitate or inhibit pain[42,43]. Endogenous analgesia refers to systems that produce the latter. These systems involve a network that includes higher cortical (e.g., anterior cingulate cortex) and subcortical regions (e.g., the amygdala, hypothalamus) that project to brainstem nuclei (periaqueductal gray and raphe nuclei) that send projections to the dorsal horn [44] These systems can be modulated by a number of factors including stress, pain and the placebo response [42].

A number of studies have used fMRI to investigate endogenous modulation of pain (Table 4) and map circuits involved in CNS systems that can alter decrease or increase pain [45-47]. Most of these studies involved attention or distraction to modulate circuits. In addition, closely linked with this are studies of placebo response, evaluation of the effects of attention and expectancy, of empathetic reactions to pain in others, to producing "trickery" of the brain by sensory inputs [48].

**Table 4:** Examples of Contributions of fMRI on Endogenous Mechanisms of Pain or Analgesia

**STUDY FOCUS**	**REFERENCE**	**MAJOR FINDINGS**	**COMMENT**
**Placebo**			
Placebo	Wager et al., 2004 [49]	2 studies report that (1) placebo analgesia decreased activation in thalamus, insula aCG and (2) anticipation increased activation in prefrontal cortex	Placebo analgesia may not only decrease pain but may change the affective response to pain.
Placebo in Emotional Processing	Petrovic et al., 2005 [84]	Use of pleasant and unpleasant pictures compared with pain. The same modulatory effect is observed in "emotional" placebo and placebo analgesia (anterior cingulated, lateral orbitofrontal cortex)	Placebo is a process in reward processing.
Placebo analgesia	Bingel et al., 2006 [85]	Nineteen healthy subjects. Placebo analgesia using laser for pain stimulation shows interaction between aCG, amygdala and PAG	Interactions between emotional circuits including the aCG amygdala and affect output processing of endogenous pain control mechanisms.
**Attention**			
Attentional modulation	Tracey et al., 2002 [46]	Nine Subjects. Distraction during painful thermal stimulus. Pain ratings significantly lower with distraction with increased activation in the PAG during this condition.	Specific output of modulatory system via a well-known brain region (i.e., PAG) presumably via inputs from higher cortical regions.
Distraction	Valet et al., 2004 [47]	Stroop task used for distraction during noxious and innocuous heat stimuli. Distraction produced decreases in VAS pain intensity and unpleasantness scores. Distraction increased activation in orbitofrontal cortex, perigenual aCG, PAG and posterior thalamus.	Covariate analysis indicated that the brain may gate information by exerting a top-down effect on PAG and posterior thalamus.
Cognitive Distraction Task	Bantick et al., 2002 [86]	Stroop task used. Intermittent thermal pain applied. Distraction produced increased activation in affective region of the aCG and in the Gob, but decreased activation in pain sensory regions including the cognitive area of the aCG, insula, and thalamus.	Dissection of the effects of distraction on emotional/affective vs. sensory systems in a distraction paradigm.
Virtual Reality Distraction	Hoffman et al., 2004. [87]	Virtual reality decreased pain; both psychophysical ratings and brain activity in aCG, SI, SII, insula and thalamus.	Distraction has not been used in fMRI studies of chronic pain.
Attention to 'location' and 'unpleasantness'	Kulkarni et al., 2005 [88]	Attention to location – activity in SI and inferior parietal cortex. Attention to unpleasantness – activation reported in aCG, orbitofrontal and frontal cortex, amygdala, hypothalamus and	Study focus is on how attention can significantly modulate pain processing.
**Miscellaneous**			
Empathy pain activates affective but not sensory pain	Singer et al., 2004. [89]	Empathy evaluated by subject in magnet observing loved one receive similar painful stimulus (empathetic pain). Activation in insular, aCG, brainstem and cerebellum by both direct or empathetic pain.	Activation in affective circuits not sensory – this may be further support for affective circuits being a focus of study in chronic pain.
Pain and Social Loss	Panksepp, 2003 [90]	Evaluation of social exclusion (rejection). Activation in aCG and ventral prefrontal cortex > with exclusions	Commonality of physical pain response and emotional rejection/hurt?
Salience of Painful Stimuli	Downar et al., 2003 [91]	Sustained pain and non-painful electrical stimulation. Transient activation during 'on' and 'off' of non-painful stimuli in aCG, inferior frontal, temporoparietal regions to non-painful. Same regions in addition to thalamus and putamen showed sustained response during painful stimulus.	Basal ganglia play a role in sustained salience.
**Expectancy orAnticipation**			
Dissociation of pain from its anticipation	Ploghaus et al., 1999 [27]	Expectation of pain produced activation in medial frontal region, insula, and cerebellum. These differed from pain experience.	Emotional/cognitive evaluation of a situation can produce adaptation that can modify the experience. This has enormous implications in the clinical situation e.g., anticipating a procedure. Effects in or on chronic pain are unknown.
Expectation of Pain	Sawamoto et al., 2000 [92]	Expectation increases response to non-painful stimuli in the aCG and posterior insula.	Pain/unpleasantness and pain relief may be opponent processing using similar circuitry.
Expectation of Pain Relief	Seymour et al., 2005. [21]	Activation in the amygdala and midbrain by pain are mirrored by opposite aversion signals in the lateral orbitofrontal cortex and aCG.	
Expected vs. Experience Pain	Koyama et al., 2005. [93]	Pain intensity to expected vs. experienced pain evaluated. With increased level of expected pain, activation increased in aCG, insula, thalamus, and prefrontal cortex. Pain experience produced activation in a number of regions (partial overlap with expectancy related pain).	Expectation can modulate the actual experience.
Anticipation of pain	Porro et al., 2002 [94]	Expectation of a pain/no pain stimulus to the foot. Activations increased in contralateral SI but decreased in ipsilateral SI, aCG.	Focus on cortical system
Hypnotically induced (HI) or imagined pain	Derbyshire et al., 2004. [95]	HI pain produced activation in thalamus, aCG, I, prefrontal and parietal cortices.	Pain pathways can be activated without a noxious stimulus. This has implications for understanding CNS processing in chronic pain disorders with no specific etiology.
Expectancy using a conditioning cue	Keltner et al,. 2006 [45]	Pain intensity expectancy acts via a modulatory network that converges on the nucleus cuneiformis (nCF)	A study defining a specific modulatory pathway on this brainstem nucleus.
**Paradoxical Sensations**			
Paradoxical heat	Davis et al., 2004. [48]	When subjects perceived a painful stimulus even though the stimulus was cool or neutral, activation in the right insular cortex was observed.	Sensory inputs in the normal healthy condition can "confuse" the brain. Such insights will be helpful in understanding pain processing in chronic conditions, particularly neuropathic pain.
Prickle sensation	Davis et al., 2002. [12]	Prickle sensation using cold, produced activations present in pain, motor and sensory areas. aCG, SII, prefrontal cortex, caudate, dorsomedial thalamus, prefrontal cortex.	Definition of the utility of percept-related fMRI – i.e., importance of on-line measures of psychophysical data.
Non-dermatomal sensory deficits.	Mailis-Gagnon et al., 2003 [96]	Noxious and non-noxious stimuli were not perceived in these dermatomes (Perceived stimuli activated posterior region of the aCG, thalamus); but produced decreased signal changes in a number of cortical regions (SI, SII, parietal cortex, prefrontal cortex and rostral aCG).	Four Patients tested to evaluate nondermatomal neurosensory deficits. Another insight into evaluating complex patient groups with altered pain processing. fMRI however does provide data in support of a testable neurobiological hypothesis instead of generic labeling of such patients.
**Acupuncture**			
Electroacupuncture vs. manual acupuncture	Napadow et al., 2005 [97]	For regional responses electroacupuncture>manual>placebo. Acupuncture induced increased activations in insula and decreased activations in amygdala, hippocampus and cingulated (subgenual and retrospelenial), ventromedial prefrontal cortex. No activations were seen for tactile control stimulations.	Difficult studies because good controls are so difficult. Process of activation of limbic and paralimbic structures may nevertheless be highly important in the therapeutic effect in clinical conditions.
Activation of PAG	Liu et al., 2004 [98]	Mechanical stimulation produced activation in PAG after 20+ min of stimulation.	Activation of endogenous analgesic systems may be part of underlying effects of acupuncture.
**Pain Control**			
Controllability	Salomons et al, 2004 [99]	Control attenuated activation in anterior cingulated and insula.	Cognitive and affective control of pain has enormous implications in clinical aspects of potential painful procedures.
Immediate Control of Brain Activation and Pain	deCharms et al., 2005. [100]	Real time fMRI (rtfMRI) to train subjects to control activation in rACC. Subjects (control and chronic pain) could change activation in ACC with corresponding change in perception to noxious stimuli	First use of rtfMRI in pain.

Perhaps of greatest importance are studies of the placebo effect since there has been a significant literature in this domain from psychophysical studies [49,50]. A neurobiology and neurocircuitry were predicted for the placebo effect based on its effects on analgesia. [51]. Indeed the general circuitry of the placebo response can be applied to non-painful stimuli. These and other studies have enhanced our knowledge of the interaction of physiological pain circuits and cognitive/emotional circuits [52]. The use of imaging has now clearly established how some of these endogenous systems operate. This understanding coupled with an objective method of evaluating placebo should provide novel insights into drug development as well as the treatment of patients with acute and chronic pain.

Complementary with fMRI studies on placebo, the use of fMRI has identified neural systems involved in anxiety and fear related to pain. These two reactions to pain are important both from a neuroscience aspect. [16] as well as from practical applications of treating patients. While fear and anxiety have been considered to have different effects on neural processing [53], recent fMRI studies have begun to explore this issue [54].

The innate nature of the pain experience is clearly indicated by studies showing that activations in non-sensory CNS systems in an observer experiencing empathetic pain, are similar to those produced in a subject by noxious stimuli. The study of endogenous analgesia highlights the opponent systems operating in pain. In addition, fMRI studies reveal that in addition to the sensory pathways activated in endogenous analgesia and pain processing, reward/aversion circuitry is activated, with reward related to pain relief and aversion related to pain. [21]. The balance between these opponent systems may be crucial in determining the overall sensory and emotional experience of pain in chronic pain states.

fMRI has also been applied to exploring the neurobiology of acupuncture, which is believed to activate endogenous analgesic mechanisms [55]. Such studies have predominantly been in healthy subjects using experimental pain. The evidence for the benefit of acupuncture for clinical studies has been mixed. For example, recent studies in migraine patients has indicated that acupuncture may be no more effective than sham acupuncture in reducing migraine headaches, although both are more effective than no intervention. Such studies need to be repeated, but raise questions as to how acupuncture works (for example, through activation of diffuse noxious inhibitory controls or activation of endogenous systems through expectancy etc.) [56]. In carefully devised studies, defining brain circuits involved in expectancy, treatment etc. may help provide a more objective evaluation of such interventions.

## fMRI Studies of Analgesics

fMRI is also being applied to the evaluation of analgesics (pharmacological MRI or phMRI). [57]. Examples are provided in Table 5.

**Table 5:** Examples of Contributions of fMRI on Analgesics

**STUDY FOCUS**	**REFERENCE**	**MAJOR FINDINGS**	**COMMENT**
**Opioids**			
Remifentanil(short acting μ opioid)	Wise et al., 2004. [101]	Activation measured in insula to noxious stimulus in subjects receiving Rx.	Study evaluates time course and half life of action of Rx in brain region.
Remifentanil	Wise et al., 2002 [102]	Activation in brain regions to noxious heat stimulus in patients receiving Rx vs. saline. Rx produced decrease in level of pain activation in insula cortex.	First study to Evaluate CNS effects on stimulus; this allowed for extraction of regions most affected by the Rx.
NaloxoneOpioid (μ antagonist)	Borras et al., 2004. [59]	10 subjects. Direct drug effects indicated increased in aCG, prefrontal cortex, hippocampus and entorrhinal cortex. Post infusion painful heat produced increase in activation (correlated with psychophysical effects).	First study on opioids to address direct CNS effects. Naloxone is a drug that has no cognitive effects in normal healthy volunteers.
Morphine (μ agonist)	Becerra 2006. [58]	Low dose morphine produces changes in reward (SLEA, NAc, GOb) endogenous analgesia (PAG), and hypnotic circuits.	fMRI of the effects of morphine on CNS circuits – indicating the use of this approach to define specific circuitry activated by a drug.
**Antiseizure meds**			
Gabapentin	Ianetti et al., 2005 [41]	Healthy volunteers. In a capsaicin model, single dose gabapentin has an antinociceptive effect but a stronger antihyperalgesic effect: the study indicates that the drug is more effective in the sensitized state.	An excellent example of the application of fMRI to drug evaluation in dissecting the value of a model with potential clinical relevance.
**Anti-inflammatory**			
Cyclogoxygenase-2 inhibitor (Cox 2)	Baliki et al., 2005 [61]	Patient with psoriatic arthritis. Single subject evaluation. Activation induced by palpating joints included thalamus, insula, SI, SII, aCG. Rx produced decrease at 1 h.	An example of specific application on evaluating efficacy of Rx. No drug site of action could be detected in this study.
**Antidepressant**			
Amitriptyline	Morgan et al., 2005 [62]	Evaluation of rectal pain in patients with irritable bowel syndrome. Rx produced a decrease in activation in aCG, parietal association cortex.	Another example of how the focus on efficacy (CNS) of drugs may be evaluated.

Analgesic effects on brain systems or neural circuits (stimulus independent) – Many analgesics have direct CNS effects, and very little is known about how they act on the human brain. Such studies are most often performed in healthy volunteers. Here the direct effect of administration of a drug is observed without any stimulus paradigm. These types of studies allow for the interrogation of effects that may not be obvious (e.g., subcortical, subconscious), for integration of how drugs may have a role on intact brain systems that still may be the case in the chronic pain state, and for the evaluation of potential side effects of drugs. Our naloxone and morphine studies (see Table 4; [58,59]) have taken this approach and indicate the ability to evaluate direct drug effects even when there are no obvious psychophysical effects (naloxone) or well-described side effect profiles (morphine) that can be evaluated based on circuit activation (e.g., reward, sedation or analgesic circuits). [59]. The ability to define specific differences across classes of drugs (e.g., antidepressants, membrane stabilizers, opioids) may not only help focus on common areas of potential mechanisms but also provide information within different drug classes (e.g., antidepressants – tricyclics vs. serotonin norepinehprine reuptake inhibitors). Advances in this domain should lead to use of standardized fMRI trials for early phase evaluation of pharmacotherapies for pain [60].

Analgesic effects on acute or chronic pain (stimulus-dependent) – In this group, the effect of the drug is evaluated in subjects usually following an applied painful stimulus. A few examples of this type of approach include the studies of cyclooxygenase (cox) inhibitors [61] and amitriptyline [62] in chronic pain conditions and the effects of drugs on capsaicin-induced hyperalgesia (see Table 5). These approaches show that pharmacological evaluation of the CNS effects of drugs is possible, suggesting that fMRI can be used for objective assessments of drug efficacy; until now, all assessments of analgesic efficacy relied on subjective psychophysical measures.

## Conclusion

A revolution in the application of a relatively new technology, fMRI, to the field of pain and analgesia is upon us. Within the next half decade, we should begin to see direct benefits in the clinical setting that could range from (a) use of fMRI to evaluate/diagnose a pain condition; (b) use of fMRI to evaluate drug efficacy in responders vs. non-responders; (c) use of fMRI to evaluate novel drug efficacy (the latter will be driven predominantly by the pharmaceutical industry) and (d) use of fMRI to provide new insights into the mechanisms of endogenous 'pain systems'. We believe there is good reason to expect that the contribution of this technology together with advances in other neurosciences will help transition the state of current pain therapy from 'o me miserum!' ('o woe is me!') to more optimistic states for both patient and clinician 'semper aliqud novii' ('always something new' .... and better/useful). We believe there is good reason to expect that the contribution of this technology together with advances in other neurosciences will significantly advance therapies for chronic pain and alleviate physical and emotional suffering for the many individuals living with this disease.

## Abbreviation List

aCG – anterior cingulated cortex; CNS – central nervous system; CRPS – complex regional pain syndrome; DNIC – Diffuse noxious inhibitory Controls; GOb – Orbitorfrontal cortex; Hi – Hippocampus; I – Insula; IBS – irritable bowel syndrome; NAc – nucleus accumbens; nCF – cuneiform nucleus; P – putamen; PAG – periaqueductal gray; pCG – posterior cingulated cortex; Rx – treatment; SCI – Spinal cord injury; SI – primary somatosensory cortex; SII – secondary somatosensory cortex; SLEA – sublenticular extended amygdala; SMP – sympathetically maintained pain; TG – trigeminal ganglion; TN – trigeminal nucleus; VAS – visual analogue scale; VMpo – ventromedial nucleus;

## References

[B1] Rowbotham M, Harden N, Stacey B, Bernstein P, Magnus-Miller L (1998). Gabapentin for the treatment of postherpetic neuralgia: a randomized controlled trial.[see comment]. JAMA.

[B2] Lesser H, Sharma U, LaMoreaux L, Poole RM (2004). Pregabalin relieves symptoms of painful diabetic neuropathy: a randomized controlled trial. Neurology.

[B3] Gilron I, Bailey JM, Tu D, Holden RR, Weaver DF, Houlden RL (2005). Morphine, gabapentin, or their combination for neuropathic pain. N Engl J Med.

[B4] Gilron I, Max MB (2005). Combination pharmacotherapy for neuropathic pain: current evidence and future directions. Expert Rev Neurother.

[B5] Woolf CJ, Borsook D, Koltzenburg M, C. Bountra RMWKS (2004). Mechanism-based classifications of pain and analgesic drug discovery. Pain: Current Understanding, Emerging Therpaies, and Novel Approaches to Drug Discovery.

[B6] Gracely RH, Geisser ME, Giesecke T, Grant MA, Petzke F, Williams DA, Clauw DJ (2004). Pain catastrophizing and neural responses to pain among persons with fibromyalgia. Brain.

[B7] Tracey I (2001). Prospects for human pharmacological functional magnetic resonance imaging (phMRI). J Clin Pharmacol.

[B8] Borsook D, Ploghaus A, Becerra L (2002). Utilizing brain imaging for analgesic drug development. Curr Opin Investig Drugs.

[B9] Logothetis NK (2002). The neural basis of the blood-oxygen-level-dependent functional magnetic resonance imaging signal. Philos Trans R Soc Lond B Biol Sci.

[B10] Becerra LR, Breiter HC, Stojanovic M, Fishman S, Edwards A, Comite AR, Gonzalez RG, Borsook D (1999). Human brain activation under controlled thermal stimulation and habituation to noxious heat: an fMRI study. Magn Reson Med.

[B11] Davis KD, Kwan CL, Crawley AP, Mikulis DJ (1998). Event-related fMRI of pain: entering a new era in imaging pain. Neuroreport.

[B12] Davis KD, Pope GE, Crawley AP, Mikulis DJ (2002). Neural correlates of prickle sensation: a percept-related fMRI study. Nat Neurosci.

[B13] Foss JM, Apkarian AV, Chialvo DR (2006). Dynamics of pain: fractal dimension of temporal variability of spontaneous pain differentiates between pain States. J Neurophysiol.

[B14] Borsook D, Becerra L (2005). Functional imaging of pain and analgesia--a valid diagnostic tool?. Pain.

[B15] Melzack R, Casey KL, Kenshalo DR (1968). Sensory, motivational, and central control determinants of pain. The Skin Senses.

[B16] Borsook D, Becerra L, Carlezon W, Shaw M, Renshaw P, Elman I, Levine JA (2005). Reward-aversion circuitry in analgesia and pain: Implications for psychiatric disorders.. European Journal of Pain.

[B17] Becerra L, Breiter HC, Wise R, Gonzalez RG, Borsook D (2001). Reward circuitry activation by noxious thermal stimuli. Neuron.

[B18] Aharon I, Becerraa L, Chabris CF, Borsooka D (2006). Noxious heat induces fMRI activation in two anatomically distinct clusters within the nucleus accumbens. Neurosci Lett.

[B19] Brooks JC, Zambreanu L, Godinez A, Craig AD, Tracey I (2005). Somatotopic organisation of the human insula to painful heat studied with high resolution functional imaging. Neuroimage.

[B20] DaSilva AF, Becerra L, Makris N, Strassman AM, Gonzalez RG, Geatrakis N, Borsook D (2002). Somatotopic activation in the human trigeminal pain pathway. J Neurosci.

[B21] Seymour B, O'Doherty JP, Koltzenburg M, Wiech K, Frackowiak R, Friston K, Dolan R (2005). Opponent appetitive-aversive neural processes underlie predictive learning of pain relief. Nat Neurosci.

[B22] Salvador R, Suckling J, Coleman MR, Pickard JD, Menon D, Bullmore E (2005). Neurophysiological architecture of functional magnetic resonance images of human brain. Cereb Cortex.

[B23] Horwitz B, Warner B, Fitzer J, Tagamets MA, Husain FT, Long TW (2005). Investigating the neural basis for functional and effective connectivity. Application to fMRI. Philos Trans R Soc Lond B Biol Sci.

[B24] Thirion B, Flandin G, Pinel P, Roche A, Ciuciu P, Poline JB (2005). Dealing with the shortcomings of spatial normalization: Multi-subject parcellation of fMRI datasets. Hum Brain Mapp.

[B25] Woldorff MG, Hazlett CJ, Fichtenholtz HM, Weissman DH, Dale AM, Song AW (2004). Functional parcellation of attentional control regions of the brain. J Cogn Neurosci.

[B26] Johansen-Berg H, Behrens TE, Sillery E, Ciccarelli O, Thompson AJ, Smith SM, Matthews PM (2005). Functional-anatomical validation and individual variation of diffusion tractography-based segmentation of the human thalamus. Cereb Cortex.

[B27] Ploghaus A, Tracey I, Gati JS, Clare S, Menon RS, Matthews PM, Rawlins JN (1999). Dissociating pain from its anticipation in the human brain. Science.

[B28] Ploghaus A, Narain C, Beckmann CF, Clare S, Bantick S, Wise R, Matthews PM, Rawlins JN, Tracey I (2001). Exacerbation of pain by anxiety is associated with activity in a hippocampal network. J Neurosci.

[B29] Ploghaus A, Becerra L, Borras C, Borsook D (2003). Neural circuitry underlying pain modulation: expectation, hypnosis, placebo. Trends Cogn Sci.

[B30] Apkarian AV, Bushnell MC, Treede RD, Zubieta JK (2005). Human brain mechanisms of pain perception and regulation in health and disease. Eur J Pain.

[B31] Giesecke T, Gracely RH, Grant MA, Nachemson A, Petzke F, Williams DA, Clauw DJ (2004). Evidence of augmented central pain processing in idiopathic chronic low back pain. Arthritis Rheum.

[B32] Verne GN, Himes NC, Robinson ME, Gopinath KS, Briggs RW, Crosson B, Price DD (2003). Central representation of visceral and cutaneous hypersensitivity in the irritable bowel syndrome. Pain.

[B33] Becerra L, Pendse G, Korn J, Shaw M, Gostic JM, Sherman S, Gostic R, Borsook D (2005). Dissecting drug efficacy for neuropathic pain in healthy subjects.: November..

[B34] Apkarian AV, Sosa Y, Sonty S, Levy RM, Harden RN, Parrish TB, Gitelman DR (2004). Chronic back pain is associated with decreased prefrontal and thalamic gray matter density. J Neurosci.

[B35] Klein T, Magerl W, Rolke R, Treede RD (2005). Human surrogate models of neuropathic pain. Pain.

[B36] Blackburn-Munro G (2004). Pain-like behaviours in animals - how human are they?. Trends Pharmacol Sci.

[B37] Mogil JS, Crager SE (2004). What should we be measuring in behavioral studies of chronic pain in animals?. Pain.

[B38] LaMotte RH, Lundberg LE, Torebjork HE (1992). Pain, hyperalgesia and activity in nociceptive C units in humans after intradermal injection of capsaicin. J Physiol.

[B39] Maihofner C, Handwerker HO, Neundorfer B, Birklein F (2004). Cortical reorganization during recovery from complex regional pain syndrome. Neurology.

[B40] Maihofner C, Handwerker HO (2005). Differential coding of hyperalgesia in the human brain: a functional MRI study. Neuroimage.

[B41] Iannetti GD, Zambreanu L, Wise RG, Buchanan TJ, Huggins JP, Smart TS, Vennart W, Tracey I (2005). Pharmacological modulation of pain-related brain activity during normal and central sensitization states in humans. Proc Natl Acad Sci U S A.

[B42] Fields HL (2000). Pain modulation: expectation, opioid analgesia and virtual pain. Prog Brain Res.

[B43] Suzuki R, Rygh LJ, Dickenson AH (2004). Bad news from the brain: descending 5-HT pathways that control spinal pain processing. Trends Pharmacol Sci.

[B44] Millan MJ (2002). Descending control of pain. Prog Neurobiol.

[B45] Keltner JR, Furst A, Fan C, Redfern R, Inglis B, Fields HL (2006). Isolating the modulatory effect of expectation on pain transmission: a functional magnetic resonance imaging study. J Neurosci.

[B46] Tracey I, Ploghaus A, Gati JS, Clare S, Smith S, Menon RS, Matthews PM (2002). Imaging attentional modulation of pain in the periaqueductal gray in humans. J Neurosci.

[B47] Valet M, Sprenger T, Boecker H, Willoch F, Rummeny E, Conrad B, Erhard P, Tolle TR (2004). Distraction modulates connectivity of the cingulo-frontal cortex and the midbrain during pain--an fMRI analysis. Pain.

[B48] Davis KD, Pope GE, Crawley AP, Mikulis DJ (2004). Perceptual illusion of "paradoxical heat" engages the insular cortex. J Neurophysiol.

[B49] Wager TD, Rilling JK, Smith EE, Sokolik A, Casey KL, Davidson RJ, Kosslyn SM, Rose RM, Cohen JD (2004). Placebo-induced changes in FMRI in the anticipation and experience of pain. Science.

[B50] Lorenz J, Hauck M, Paur RC, Nakamura Y, Zimmermann R, Bromm B, Engel AK (2005). Cortical correlates of false expectations during pain intensity judgments--a possible manifestation of placebo/nocebo cognitions. Brain Behav Immun.

[B51] Fields HL, Levine JD (1981). Biology of placebo analgesia. Am J Med.

[B52] Kong J, White NS, Kwong KK, Vangel MG, Rosman IS, Gracely RH, Gollub RL (2005). Using fMRI to dissociate sensory encoding from cognitive evaluation of heat pain intensity. Hum Brain Mapp.

[B53] Gray JAMNN, N.J. Mackintosh TSATJLMGDSLW (2000). The Neuropsychology of Anxiety: An Enquiry into the Functions of the Septo-Hippocampal System. Oxford Psychology Series.

[B54] Ochsner KN, Ludlow DH, Knierim K, Hanelin J, Ramachandran T, Glover GC, Mackey SC (2006). Neural correlates of individual differences in pain-related fear and anxiety. Pain.

[B55] Ma SX (2004). Neurobiology of Acupuncture: Toward CAM. Evid Based Complement Alternat Med.

[B56] Sandkuhler J (1996). The organization and function of endogenous antinociceptive systems. Prog Neurobiol.

[B57] Honey G, Bullmore E (2004). Human pharmacological MRI. Trends Pharmacol Sci.

[B58] Becerra L, Harter K, Gonzalez RG, Borsook D (2006). Functional magnetic resonance imaging measures of the effects of morphine on central nervous system circuitry in opioid-naive healthy volunteers. Anesth Analg.

[B59] Borras MC, Becerra L, Ploghaus A, Gostic JM, DaSilva A, Gonzalez RG, Borsook D (2004). fMRI measurement of CNS responses to naloxone infusion and subsequent mild noxious thermal stimuli in healthy volunteers. J Neurophysiol.

[B60] Borsook D, Becerra L, Hargreaves RA I spy fMRI: To be or not to be in drug development. Nature Reviews Drug Discovery.

[B61] Baliki M, Katz J, Chialvo DR, Apkarian AV (2005). Single subject pharmacological-MRI (phMRI) study: modulation of brain activity of psoriatic arthritis pain by cyclooxygenase-2 inhibitor. Mol Pain.

[B62] Morgan V, Pickens D, Gautam S, Kessler R, Mertz H (2005). Amitriptyline reduces rectal pain related activation of the anterior cingulate cortex in patients with irritable bowel syndrome. Gut.

[B63] Borsook D, DaSilva AF, Ploghaus A, Becerra L (2003). Specific and somatotopic functional magnetic resonance imaging activation in the trigeminal ganglion by brush and noxious heat. J Neurosci.

[B64] Fulbright RK, Troche CJ, Skudlarski P, Gore JC, Wexler BE (2001). Functional MR imaging of regional brain activation associated with the affective experience of pain. AJR Am J Roentgenol.

[B65] Dunckley P, Wise RG, Fairhurst M, Hobden P, Aziz Q, Chang L, Tracey I (2005). A comparison of visceral and somatic pain processing in the human brainstem using functional magnetic resonance imaging. J Neurosci.

[B66] Bingel U, Glascher J, Weiller C, Buchel C (2004). Somatotopic representation of nociceptive information in the putamen: an event-related fMRI study. Cereb Cortex.

[B67] Ploghaus A, Tracey I, Clare S, Gati JS, Rawlins JN, Matthews PM (2000). Learning about pain: the neural substrate of the prediction error for aversive events. Proc Natl Acad Sci U S A.

[B68] Rolls ET, O'Doherty J, Kringelbach ML, Francis S, Bowtell R, McGlone F (2003). Representations of pleasant and painful touch in the human orbitofrontal and cingulate cortices. Cereb Cortex.

[B69] Peyron R, Schneider F, Faillenot I, Convers P, Barral FG, Garcia-Larrea L, Laurent B (2004). An fMRI study of cortical representation of mechanical allodynia in patients with neuropathic pain. Neurology.

[B70] Becerra L, Morris S, Bazes S, Gostic R, Sherman S, Gostic J, Pendse G, Moulton E, Scrivani S, Keith D, Chizh B, Borsook D (2006). Trigeminal Neuropathic Pain Alters Responses in CNS Circuits to Mechanical (brush) and Thermal (cold and heat) Stimuli. J Neurosci.

[B71] Nicotra A, Critchley HD, Mathias CJ, Dolan RJ (2005). Emotional and autonomic consequences of spinal cord injury explored using functional brain imaging. Brain.

[B72] Villemure C, Wassimi S, Bennett GJ, Shir Y, Bushnell MC (2006). Unpleasant odors increase pain processing in a patient with neuropathic pain: Psychophysical and fMRI investigation. Pain.

[B73] Lebel A, Becerra L, Waring M, Morris S, Pendse G, Grant E, Moulton E, Berde C, Borsook D (2005). fMRI of mechanical allodynia in children with complex regional (leg) pain syndrome (CRPS).. Soc Neuroscience.

[B74] Apkarian AV, Thomas PS, Krauss BR, Szeverenyi NM (2001). Prefrontal cortical hyperactivity in patients with sympathetically mediated chronic pain. Neurosci Lett.

[B75] Cook DB, Lange G, Ciccone DS, Liu WC, Steffener J, Natelson BH (2004). Functional imaging of pain in patients with primary fibromyalgia. J Rheumatol.

[B76] Kwan CL, Diamant NE, Pope G, Mikula K, Mikulis DJ, Davis KD (2005). Abnormal forebrain activity in functional bowel disorder patients with chronic pain. Neurology.

[B77] Wilder-Smith CH, Schindler D, Lovblad K, Redmond SM, Nirkko A (2004). Brain functional magnetic resonance imaging of rectal pain and activation of endogenous inhibitory mechanisms in irritable bowel syndrome patient subgroups and healthy controls. Gut.

[B78] Mertz H, Morgan V, Tanner G, Pickens D, Price R, Shyr Y, Kessler R (2000). Regional cerebral activation in irritable bowel syndrome and control subjects with painful and nonpainful rectal distention. Gastroenterology.

[B79] Bonaz B, Baciu M, Papillon E, Bost R, Gueddah N, Le Bas JF, Fournet J, Segebarth C (2002). Central processing of rectal pain in patients with irritable bowel syndrome: an fMRI study. Am J Gastroenterol.

[B80] Pukall CF, Strigo IA, Binik YM, Amsel R, Khalife S, Bushnell MC (2005). Neural correlates of painful genital touch in women with vulvar vestibulitis syndrome. Pain.

[B81] Baron R, Baron Y, Disbrow E, Roberts TP (1999). Brain processing of capsaicin-induced secondary hyperalgesia: a functional MRI study. Neurology.

[B82] Zambreanu L, Wise RG, Brooks JC, Iannetti GD, Tracey I (2005). A role for the brainstem in central sensitisation in humans. Evidence from functional magnetic resonance imaging. Pain.

[B83] Wiech K, Seymour B, Kalisch R, Stephan KE, Koltzenburg M, Driver J, Dolan RJ (2005). Modulation of pain processing in hyperalgesia by cognitive demand. Neuroimage.

[B84] Petrovic P, Dietrich T, Fransson P, Andersson J, Carlsson K, Ingvar M (2005). Placebo in emotional processing--induced expectations of anxiety relief activate a generalized modulatory network. Neuron.

[B85] Bingel U, Lorenz J, Schoell E, Weiller C, Buchel C (2006). Mechanisms of placebo analgesia: rACC recruitment of a subcortical antinociceptive network. Pain.

[B86] Bantick SJ, Wise RG, Ploghaus A, Clare S, Smith SM, Tracey I (2002). Imaging how attention modulates pain in humans using functional MRI. Brain.

[B87] Hoffman HG, Richards TL, Coda B, Bills AR, Blough D, Richards AL, Sharar SR (2004). Modulation of thermal pain-related brain activity with virtual reality: evidence from fMRI. Neuroreport.

[B88] Kulkarni B, Bentley DE, Elliott R, Youell P, Watson A, Derbyshire SW, Frackowiak RS, Friston KJ, Jones AK (2005). Attention to pain localization and unpleasantness discriminates the functions of the medial and lateral pain systems. Eur J Neurosci.

[B89] Singer T, Seymour B, O'Doherty J, Kaube H, Dolan RJ, Frith CD (2004). Empathy for pain involves the affective but not sensory components of pain. Science.

[B90] Panksepp J (2003). Neuroscience. Feeling the pain of social loss. Science.

[B91] Downar J, Mikulis DJ, Davis KD (2003). Neural correlates of the prolonged salience of painful stimulation. Neuroimage.

[B92] Sawamoto N, Honda M, Okada T, Hanakawa T, Kanda M, Fukuyama H, Konishi J, Shibasaki H (2000). Expectation of pain enhances responses to nonpainful somatosensory stimulation in the anterior cingulate cortex and parietal operculum/posterior insula: an event-related functional magnetic resonance imaging study. J Neurosci.

[B93] Koyama T, McHaffie JG, Laurienti PJ, Coghill RC (2005). The subjective experience of pain: where expectations become reality. Proc Natl Acad Sci U S A.

[B94] Porro CA, Baraldi P, Pagnoni G, Serafini M, Facchin P, Maieron M, Nichelli P (2002). Does anticipation of pain affect cortical nociceptive systems?. J Neurosci.

[B95] Derbyshire SW, Whalley MG, Stenger VA, Oakley DA (2004). Cerebral activation during hypnotically induced and imagined pain. Neuroimage.

[B96] Mailis-Gagnon A, Giannoylis I, Downar J, Kwan CL, Mikulis DJ, Crawley AP, Nicholson K, Davis KD (2003). Altered central somatosensory processing in chronic pain patients with "hysterical" anesthesia. Neurology.

[B97] Napadow V, Makris N, Liu J, Kettner NW, Kwong KK, Hui KK (2005). Effects of electroacupuncture versus manual acupuncture on the human brain as measured by fMRI. Hum Brain Mapp.

[B98] Liu WC, Feldman SC, Cook DB, Hung DL, Xu T, Kalnin AJ, Komisaruk BR (2004). fMRI study of acupuncture-induced periaqueductal gray activity in humans. Neuroreport.

[B99] Salomons TV, Johnstone T, Backonja MM, Davidson RJ (2004). Perceived controllability modulates the neural response to pain. J Neurosci.

[B100] deCharms RC, Maeda F, Glover GH, Ludlow D, Pauly JM, Soneji D, Gabrieli JD, Mackey SC (2005). Control over brain activation and pain learned by using real-time functional MRI. Proc Natl Acad Sci U S A.

[B101] Wise RG, Williams P, Tracey I (2004). Using fMRI to quantify the time dependence of remifentanil analgesia in the human brain. Neuropsychopharmacology.

[B102] Wise RG, Rogers R, Painter D, Bantick S, Ploghaus A, Williams P, Rapeport G, Tracey I (2002). Combining fMRI with a pharmacokinetic model to determine which brain areas activated by painful stimulation are specifically modulated by remifentanil. Neuroimage.

